# Educational disparities in nasopharyngeal carcinoma survival: Temporal trends and mediating effects of clinical factors

**DOI:** 10.1002/ctm2.134

**Published:** 2020-07-22

**Authors:** Wang‐Zhong Li, Guo‐Ying Liu, Shu‐Hui Lv, Sen‐Kui Xu, Hu Liang, Kui‐Yuan Liu, Meng‐Yun Qiang, Xi Chen, Xiang Guo, Xing Lv, Wei‐Xiong Xia, Yan‐Qun Xiang

**Affiliations:** ^1^ State Key Laboratory of Oncology in South China Collaborative Innovation Center for Cancer Medicine Guangdong Key Laboratory of Nasopharyngeal Carcinoma Diagnosis and Therapy Sun Yat‐Sen University Cancer Center Guangzhou China; ^2^ Department of Nasopharyngeal Carcinoma Sun Yat‐Sen University Cancer Center Guangzhou China; ^3^ Department of Radiation Oncology Sun Yat‐Sen University Cancer Center Guangzhou China

Dear Editor,

Previous studies conducted in various settings have reported the strong association of socioeconomic status (SES) with cancer risk and cancer mortality.^1^, ^2^, ^3^ Low SES has been associated with increased incidence of malignancies and worse survival. Educational level (EL) is considered as a critical component of SES and could be a better indicator linked to social position.^4^, ^5^ EL is closely correlated with behavioral factors, comorbidity burden, health awareness, and accessibility to the health care system, which may have direct or indirect effects on prognosis of cancer.^6^, ^7^, ^8^ Furthermore, factors related to education may influence cancer in various way, including tumor stage at diagnosis, pathologic features, choice of treatment, and eventually survival.^7^, ^8^, ^9^, ^10^ The impact of educational inequalities has well established for most malignant tumors.^6^, ^7^, ^8^, ^9^, ^10^ However, there has been no study to date examining the effect of educational disparities on the survival of patients with nasopharyngeal carcinoma (NPC). In this study, we sought to examine the effect of EL on NPC survival and evaluate its temporal trend. We also estimated the mediating effects of tumor characteristics, lifestyle factors, sociodemographic factors, and treatment modality on educational disparities in NPC survival.

A total of 9346 eligible patients with newly diagnosed NPC were included in analysis. The patient characteristics stratified by EL are listed in Table S1. EL was classified into three categories according to the International Standard Classification of Education (ISCED, 2011 version) as follows: low (ISCED 0‐2: less than primary education and lower secondary education), medium (ISCED 3‐4: upper secondary education), and high (ISCED 5‐6: tertiary education). Among them, 45.8% of patients (N = 4284) had low EL, 33.0% (N = 3089) had medium EL, and 21.1% (N = 1973) had high EL. Compared to lower educated patients, patients with high EL were more likely associated with favorable characteristics. The median follow‐up time was 8.2 years. Crude survival rates stratified by EL revealed significant survival differences across all three groups (Figure 1A, all *P* < .001). A higher EL conferred a better prognosis. The estimated 10‐year cancer‐specific survival (CSS) was superior in high‐educated patients (77.7%) compared with 74.3% and 68.2% in medium and low EL, respectively. Univariable Cox regression analyses showed that EL (Hazard ratios [HRs]: high vs low, .646; 95% confidence interval [CI], .575‐.726, *P* < .001; medium vs low, .796; 95% CI, .724‐.875, *P* < .001; Table S2) was significantly associated with CSS. Multivariable analysis revealed that EL was a significant independent factor. HRs for death resulting from NPC decreased with higher education (adjusted HRs: high vs low, .859; 95% CI, .761‐.969, *P* = .014; medium vs low, .891; 95% CI, .809‐.981, *P* = .019; Table S2).

**FIGURE 1:**
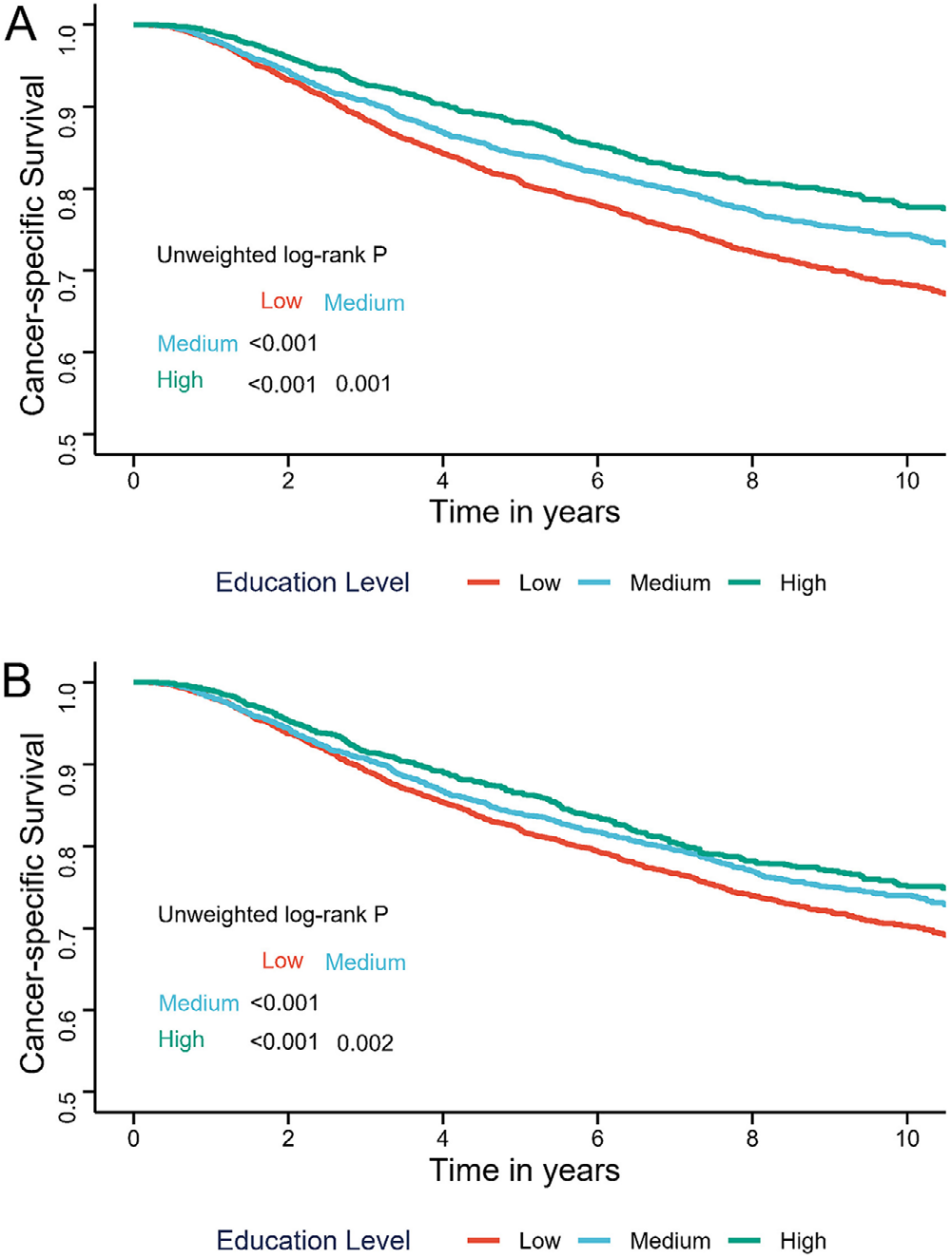
Cancer‐specific survival curves with log‐rank test stratified by education level: crude (A) and inverse probability of treatment weighting (IPTW) adjusted (B)

EL as an independent prognostic factor was further confirmed through the inverse probability of treatment weighting (IPTW)‐adjusted K‐M survival analysis and Cox regression model. The unweighted and weighted patient characteristics of the study population, stratified by EL, are listed in Table S1. In our study, 1000 model iterations can sufficiently optimize the balance statistics when estimating the weights (Figure S1A). The boxplot demonstrated that there was a substantial overlap in the total spread of propensity scores and the median propensity score by different education groups (Figure S1B). Following IPTW adjustment, most of the standardized differences were less than 10% (Figure S1C), and most of the weighted *t*‐test or chi‐square statistic *P*‐values for differences between three education groups were not significant (Figure S1D), indicating that distribution of groups was comparable. IPTW‐adjusted Kaplan‐Meier analysis showed that differences across three educational groups remained statistically significant (Figure 1B, all *P* < .05). In IPTW‐adjusted Cox regression model, minor modification of HRs was observed but they kept statistically significant (IPTW‐adjusted HR: high vs low, .830; 95% CI, .724‐.949, *P* = .006; medium vs low, .898; 95% CI, .884‐.991, *P* = .032; Table S2).

There was a significant nonlinear effect of the year of diagnosis on NPC survival in patients with high EL (*P* = .003). The restricted cubic spline plots showed that the log relative hazard of NPC‐specific mortality decreased for each educational category from 2000 to 2011 (Figure 2A‐C). As a result, the 3‐ and 5‐year CSS rates improved over time in all three education groups (Figure 2D). The pace of decreasing HR over time, however, was steeper for patients with high EL than patients with low or medium EL (Figure 2D). The protective effect of high EL became more prominent for the most recent years (HR—2000‐2003: .730 [95% CI, .605‐.880]; 2004‐2007: .699 [95% CI, .544‐.899]; 2008‐2011: .638 [95% CI, .529‐.796]; Figure 2D). As a result, the survival disparities between low and high EL became more evident in recent years.

**FIGURE 2:**
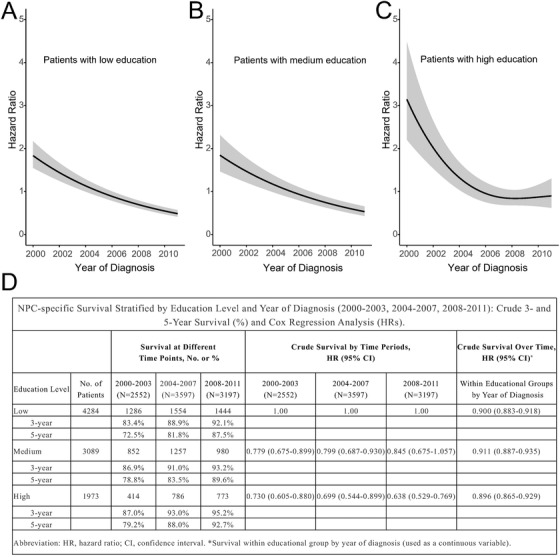
Temporal trends of the hazard ratio of NPC‐specific death during 2000‐2011 in three different educational groups (A‐C), and NPC‐specific survival stratified by education level and year of diagnosis (2000‐2003, 2004‐2007, and 2008‐2011): crude 3‐ and 5‐year survival and cox regression analysis (D)

To explore the underlying paths linking EL to NPC‐specific mortality, we explored the role of clinical characteristics, sociodemographic characteristics, and treatment factors as mediators to the observed educational disparities. We observed that 57.1% (95% CI, 36.0‐79.7%, Figure S2A) of the excess NPC mortality among patients with medium EL could be attributed to the mediators, including age at diagnosis (39.0%), radiotherapy (RT) technique (13.1%), clinical stage (6.9%), tumor stage (5.4%), body mass index (BMI) (5.1%), and gender (–12.9%). Among patients with high EL, the significant mediators included age at diagnosis (28.0%, Figure S2B), RT technique (16.9%), clinical stage (12.5%), tumor stage (9.4%), node stage (7.5%), BMI (5.14.6%), and gender (–7.0%). The total proportion mediated through these seven factors was about 71.5% (95% CI, 55.7‐91.3%).

Our database is among the largest available database of NPC patients that includes sociodemographic information, clinical characteristics, lifestyle factors, and treatment modalities. In this study involving 9346 participants, we found that it is individual EL rather than other SES a significant independent prognostic factor in NPC. Although the NPC death rate decreased in all educational groups in recent years, educational disparities in NPC mortality between low‐ and high‐educated patients widened. Multiple mediation analyses show that factors such as age at diagnosis, stage at diagnosis, RT technique, BMI, and gender were significant mediators contributed to educational disparities in NPC survival. Further study is necessary to explore other unidentified risk factors that might interact with EL and find out possible points of intervention that may reduce educational disparities.

## AUTHOR CONTRIBUTIONS

YQ Xiang, WX Xia, and WZ Li designed the study. WZ Li, GY Liu, and SK Xu developed the methodology of the study. WZ Li, GY Liu, SK Xu, SH Lv, H Liang, KY Liu, MY Qiang, ZC Cai, X Chen, CX Liang, X Lv, and X Guo participated in the acquisition of data. WZ Li analyzed and interpreted the data. WZ Li wrote the manuscript. All authors reviewed and revised the manuscript.

## DATA ACCESS, RESPONSIBILITY, AND ANALYSIS

All data generated or analyzed during the present study are available via the corresponding author on reasonable request. The data are under review and will link to Research Data Deposit (http://www.researchdata.org.cn/) with a unique deposit ID.

## ETHICS APPROVAL AND CONSENT TO PARTICIPATE

The study protocol was approved by the institutional review board of Sun Yat‐Sen University Cancer Center. Informed consent was obtained from each patient before treatment.

## CONFLICT OF INTEREST

The authors declare no conflict of interest.

## Supporting information

Supporting Information
