# Supplementary material for: Educational disparities in nasopharyngeal carcinoma survival: Temporal trends and mediating effects of clinical factors
Source: Clin Transl Med. 2020 Jul 22;10(3):e134. doi: 10.1002/ctm2.134 (PMC7418802; doi:10.1002/ctm2.134)
Supplement: Supplementary file 1 — Supporting Information [file CTM2-10-e134-s001.doc]

**Supplementary Materials**

**Table S1.** Patient Characteristics Stratified by Education Level.

**Table S2**. Results of the univariable and multivariable Cox regression model.

**Figure S1**. An optimization plot for the balance statistics when estimating the propensity scores (A), a boxplot illustrating the spread of propensity scores and the median propensity score by different education groups (B), the absolute standard difference of unweighted and weighted population (C), and rank of the p−value for pretreatment variables in the unweighted and weighted population (D).

**Figure S2.** Relative effects of moderators in patients with medium (A) and high (B) education level.

| **Table S1.** Patient Characteristics Stratified by Education Level. | | | | | | | | | | |
| --- | --- | --- | --- | --- | --- | --- | --- | --- | --- | --- |
| **Characteristic** | **Overall**  **(N=9346)** | **Unweighted education level** | | |  |  | **Weighted education level** | | | |
|  |  | **Low (n=4284)** | **Medium (n=3089)** | **High (n=1973)** | ***P* value** |  | **Low** | **Medium** | **High** | ***P* value** |
| Mean age (y) ± SD | 46.2 ± 11.2 | 48.1 ± 11.0 | 45.3 ± 11.0 | 43.5 ± 11.3 | <.001 |  | 46.3 ± 11.0 | 45.9 ± 10.9 | 45.4 ± 10.9 | .238 |
| Gender |  |  |  |  | <.001 |  |  |  |  | .069 |
| Male | 7026 (75.2) | 3025 (70.6) | 2424 (78.4) | 1578 (80.0) |  |  | 74.9 | 76.5 | 76.7 |  |
| Female | 2320 (24.8) | 1259 (29.4) | 666 (21.6) | 395 (20.0) |  |  | 25.1 | 23.5 | 23.3 |  |
| Comorbidity score |  |  |  |  | .129 |  |  |  |  | .325 |
| 0 | 7193 (77.0) | 3338 (77.9) | 2355 (76.2) | 1500 (76.0) |  |  | 77.5 | 77.2 | 77.0 |  |
| ≧1 | 2153 (23.0) | 946 (22.1) | 734 (23.8) | 473 (24.0) |  |  | 22.5 | 22.8 | 23.0 |  |
| Smoking status |  |  |  |  | <.001 |  |  |  |  | .100 |
| No | 5227 (55.9) | 2348 (54.8) | 1609 (52.1) | 1270 (64.4) |  |  | 55.3 | 55.0 | 57.3 |  |
| Yes | 4119 (44.1) | 1936 (45.2) | 1480 (47.9) | 703 (35.6) |  |  | 44.7 | 45.0 | 42.7 |  |
| Drinking status |  |  |  |  | .528 |  |  |  |  | .174 |
| No | 7919 (84.7) | 3625 (84.6) | 2634 (85.3) | 1660 (84.1) |  |  | 85.0 | 85.0 | 84.8 |  |
| Yes | 1427 (15.3) | 659 (15.4) | 455 (14.7) | 313 (15.9) |  |  | 15.0 | 15.0 | 15.2 |  |
| Mean BMI (kg/m^2^) ± SD | 22.9 ± 3.2 | 22.7 ± 3.2 | 22.9 ± 3.3 | 23.2 ± 3.2 | <.001 |  | 22.8 ± 3.2 | 22.9 ± 3.1 | 22.9 ± 3.1 | .545 |
| Histology type |  |  |  |  | .384 |  |  |  |  | .388 |
| Type I | 38 (0.4) | 22 (0.5) | 11 (0.4) | 5 (0.3) |  |  | 0.4 | 0.3 | 0.3 |  |
| Type II | 494 (5.3) | 227 (5.3) | 153 (5.0) | 114 (5.8) |  |  | 5.4 | 5.1 | 5.5 |  |
| Type III | 8814 (94.3) | 4035 (94.2) | 2925 (94.7) | 1854 (94.0) |  |  | 94.2 | 94.5 | 94.2 |  |
| Tumor stage |  |  |  |  | <.001 |  |  |  |  | .092 |
| T1 | 838 (9.0) | 329 (7.7) | 257 (8.3) | 252 (12.8) |  |  | 8.6 | 8.8 | 9.4 |  |
| T2 | 2388 (25.6) | 1035 (24.2) | 812 (26.3) | 541 (27.4) |  |  | 25.2 | 25.6 | 26.4 |  |
| T3 | 3930 (42.1) | 1830 (42.7) | 1296 (42.0) | 804 (40.8) |  |  | 42.2 | 42.0 | 41.7 |  |
| T4 | 2190 (23.4) | 1090 (25.4) | 724 (23.4) | 376 (19.1) |  |  | 24.1 | 23.6 | 22.5 |  |
| Node stage |  |  |  |  | <.001 |  |  |  |  | .437 |
| N0 | 2078 (22.2) | 888 (20.7) | 679 (22.0) | 511 (25.9) |  |  | 21.8 | 22.1 | 22.3 |  |
| N1 | 3659 (39.2) | 1703 (39.8) | 1172 (37.9) | 784 (39.7) |  |  | 39.4 | 38.9 | 39.8 |  |
| N2 | 2908 (31.1) | 1370 (32.0) | 993 (321) | 545 (27.6) |  |  | 31.5 | 31.4 | 30.8 |  |
| N3 | 701 (7.5) | 323 (7.5) | 245 (7.9) | 133 (6.7) |  |  | 7.3 | 7.5 | 7.0 |  |
| Clinical stage |  |  |  |  | <.001 |  |  |  |  | .091 |
| Ⅰ | 322 (3.4) | 114 (2.7) | 93 (3.0) | 115 (5.8) |  |  | 3.0 | 3.3 | 3.6 |  |
| Ⅱ | 1755 (18.8) | 728 (17.0) | 599 (19.4) | 428 (21.7) |  |  | 18.6 | 19.0 | 18.9 |  |
| Ⅲ | 4518 (48.3) | 2095 (48.9) | 1476 (47.8) | 947 (48.0) |  |  | 48.4 | 48.1 | 49.3 |  |
| Ⅳ | 2751 (29.4) | 1347 (31.4) | 921 (29.8) | 483 (24.5) |  |  | 30.0 | 29.6 | 28.2 |  |
| RT technique |  |  |  |  | <.001 |  |  |  |  | .028 |
| 2DRT | 7042 (75.3) | 3453 (80.6) | 2318 (75.0) | 1274 (64.3) |  |  | 76.3 | 75.2 | 73.7 |  |
| IMRT | 2304 (24.7) | 831 (19.4) | 771 (25.0) | 706 (35.7) |  |  | 23.7 | 24.8 | 26.3 |  |
| Treatment modality |  |  |  |  | <.001 |  |  |  |  | .334 |
| RT alone | 2805 (30.0) | 1276 (29.8) | 921 (29.8) | 608 (30.8) |  |  | 29.7 | 29.6 | 28.7 |  |
| CCRT | 2263 (24.2) | 1006 (23.5) | 714 (23.1) | 543 (27.5) |  |  | 70.3 | 70.4 | 71.3 |  |
| ICT+CCRT | 4008 (42.9) | 1893 (44.2) | 1366 (44.2) | 749 (38.0) |  |  |  |  |  |  |
| ICT+CCRT+ACT | 270 (2.9) | 109 (2.5) | 88 (2.8) | 73 (3.7) |  |  |  |  |  |  |
| Marital status |  |  |  |  | <.001 |  |  |  |  | .206 |
| Unmarried | 8917 (95.4) | 162 (3.8) | 137 (4.4) | 130 (6.6) |  |  | 4.3 | 4.2 | 6.6 |  |
| Married | 429 (4.6) | 4122 (96.2) | 2952 (95.6) | 1843 (93.4) |  |  | 95.7 | 95.8 | 93.4 |  |
| Employment |  |  |  |  | <.001 |  |  |  |  | .006 |
| Unemployed | 817 (8.7) | 521 (12.2) | 258 (8.4) | 38 (4.7) |  |  | 9.1 | 6.4 | 1.9 |  |
| Employed | 7918 (84.7) | 3496 (81.6) | 2556 (82.7) | 1866 (94.6) |  |  | 84.8 | 85.1 | 94.6 |  |
| Retired | 611 (6.5) | 267 (6.2) | 275 (8.9) | 69 (3.5) |  |  | 6.1 | 6.4 | 3.5 |  |
| Notes: Unless otherwise noted, data are given as No. (%) in overall and unweighted cohort whereas data are given as % in weighted cohort. EL was classified into three categories according to the International Standard Classification of Education (ISCED, 2011 version) as follows: low (ISCED 0-2: less than primary education, primary and lower secondary education), medium (ISCED 3-4: upper secondary education), and high (ISCED 5-6: tertiary education). Abbreviations: SD, standard deviation; BMI, body mass index; RT, radiotherapy; 2DRT, two-dimensional radiotherapy; IMRT, intensity-modulated radiotherapy; CCRT, concurrent chemo-radiotherapy; ICT, induction chemotherapy; ACT, adjuvant chemotherapy. | | | | | | | | | | |

| **Table S2.** Results of the univariable and multivariable Cox regression model. | | | | | | |
| --- | --- | --- | --- | --- | --- | --- |
|  | **Univariable Model 1** | | **Multivariable Model** | | | |
|  |  |  | **Unweighted** **Model 2** | | **Weighted Model 3** | |
| **Variable** | **HR (95% CI)** | **P** | **HR (95% CI)** | **P** | **HR (95% CI)** | **P** |
| Age (continuous) | 1.031 (1.024 to 1.034) | <.001 | 1.030 (1.026 to 1.034) | <.001 | 1.029 (1.025 to 1.034) | <.001 |
| Gender (male) | 1.516 (1.363 to 1.687) | <.001 | 1.362 (1.202 to 1.543) | <.001 | 1.416 (1.123 to 1.637) | <.001 |
| Comorbidity score (≥1) | 1.033 (0.936 to 1.141) | .515 | 1.042 (0.943 to 1.151) | .418 | 1.051 (0.938 to 1.178) | .393 |
| Smoking status (yes) | 1.500 (1.380 to 1.630) | <.001 | 1.038 (0.938 to 1.149) | .466 | 1.004 (0.896 to 1.124) | .949 |
| Drinking status (yes) | 1.392 (1.253 to 1.548) | <.001 | 1.115 (0.996 to 1.249) | .059 | 1.048 (0.917 to 1.198) | .488 |
| BMI (continuous) | 0.958 (0.946 to 0.971) | <.001 | 0.960 (0.947 to 0.973) | <.001 | 0.958 (0.943 to 0.973) | <.001 |
| Histology type |  |  |  |  |  |  |
| Type Ⅰ | 1.000 |  | 1.000 |  | 1.000 |  |
| Type Ⅱ | 0487 (0.288 to 0.823) | .007 | 0.650 (0.384 to 1.101) | .109 | 0.794 (0.349 to 1.808) | .582 |
| Type Ⅲ | 0.526 (0.322 to 0.860) | .010 | 0.695 (0.424 to 1.139) | .149 | 0.794 (0.358 to 1.759) | .569 |
| Tumor stage |  |  |  |  |  |  |
| T1 | 1.000 |  | 1.000 |  | 1.000 |  |
| T2 | 1.685 (1.362 to 2.083) | <.001 | 1.380 (1.091 to 1.745) | .007 | 1.372 (1.060 to 1.778) | .016 |
| T3 | 2.143 (1.7748 to 2.626) | <.001 | 1.523 (1.194 to 1.941) | .001 | 1.497 (1.139 to 1.968) | .004 |
| T4 | 3.762 (3.063 to 4.619) | <.001 | 2.099 (1.479 to 2.979) | <.001 | 1.816 (1.196 to 2.757) | .005 |
| Node stage |  |  |  |  |  |  |
| N0 | 1.000 |  | 1.000 |  | 1.000 |  |
| N1 | 1.482 (1.308 to 1.679) | <.001 | 1.397 (1.227 to 1.589) | <.001 | 1.382 (1.199 to 1.591) | <.001 |
| N2 | 1.902 (1.676 to 2.158) | <.001 | 1.902 (1.648 to 2.196) | <.001 | 1.908 (1.629 to 2.234) | <.001 |
| N3 | 3.450 (2.943 to 4.045) | <.001 | 2.675 (2.035 to 3.517) | <.001 | 2.402 (1.731 to 3.333) | <.001 |
| Clinical stage |  |  |  |  |  |  |
| Ⅰ | 1.000 |  | 1.000 |  | 1.000 |  |
| Ⅱ | 2.542 (1.631 to 3.963) | <.001 | 1.658 (1.006 to 2.732) | .047 | 1.786 (1.060 to 3.010) | .029 |
| Ⅲ | 4.149 (2.692 to 6.393) | <.001 | 2.173 (1.304 to 3.621) | .002 | 2.440 (1.421 to 4.189) | .001 |
| Ⅳ | 8.157 (5.292 to 12.573) | <.001 | 2.995 (1.685 to 5.321) | <.001 | 3.895 (2.068 to 7.338) | <.001 |
| RT type (IMRT) | 0.557 (0.494 to 0.628) | <.001 | 0.616 (0.545 to 0.696) | <.001 | 0.615 (0.539 to 0.701) | <.001 |
| Chemotherapy |  |  |  |  |  |  |
| RT alone | 1.000 |  | 1.000 |  | 1.000 |  |
| CCRT | 1.000 (0.887 to 1.128) | .995 | 0.760 (0.668 to 0.865) | <.001 | 0.748 (0.648 to 0.863) | <.001 |
| ICT+CCRT | 1.353 (1.226 to 1.494) | <.001 | 0.806 (0.718 to 0.905) | .001 | 0.778 (0.681 to 0.888) | .002 |
| ICT+CCRT+ACT | 1.368 (1.072 to 1.745) | .012 | 0.909 (0.707 to 1.170) | .460 | 0.905 (0.674 to 1.217) | .510 |
| Marital status (married) | 1.302 (1.042 to 1.628) | .020 | 1.075 (0.859 to 1.346) | .528 | 1.042 (0.774 to 1.403) | .377 |
| Employment |  |  |  |  |  |  |
| Unemployed | 1.000 |  | 1.000 |  | 1.000 |  |
| Employed | 1.134 (0.959 to 1.341) | .142 | 1.145 (0.965 to 1.358) | .120 | 1.110 (0.870 to 1.416) | .402 |
| Retired | 1.664 (1.339 to 2.067) | <.001 | 1.065 (0.849 to 1.337) | .586 | 1.000 (0.810 to 1.22) | .786 |
| Education level |  |  |  |  |  |  |
| Low | 1.000 |  | 1.000 |  | 1.000 |  |
| Medium | 0.796 (0.724 to 0.875) | <.001 | 0.891 (0.809 to 0.981) | .019 | 0.898 (0.884 to 0.991) | .032 |
| High | 0.646 (0.575 to 0.726) | <.001 | 0.859 (0.761 to 0.969) | .014 | 0.830 (0.724 to 0.949) | .006 |
| Notes: Model 1 was univariable Cox regression model. Model 2 was fully adjusted multivariable Cox regression model. Model 3 was fully adjusted multivariable Cox regression model with inverse probability of treatment weighting (IPTW) incorporated.  Abbreviations: HR, hazard ratio; CI, confidence interval; BMI, body mass index; RT, radiotherapy; IMRT, intensity-modulated radiotherapy;2DRT, two-dimensional radiotherapy; CCRT, concurrent chemo-radiotherapy; ICT, induction chemotherapy; ACT, adjuvant chemotherapy. | | | | | | |


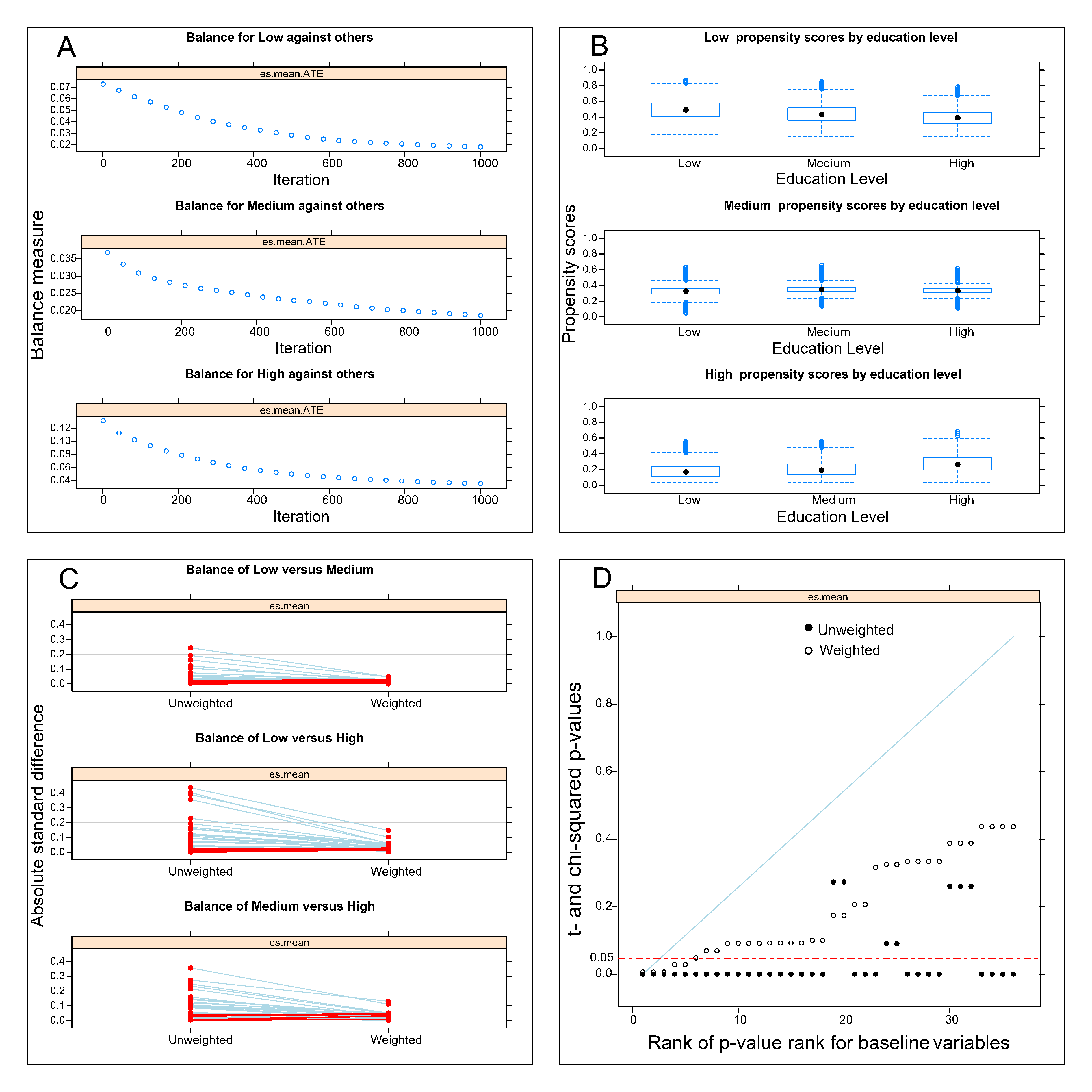


**Figure S1**. An optimization plot for the balance statistics when estimating the propensity scores (A), a boxplot illustrating the spread of propensity scores and the median propensity score by different education groups (B), the absolute standard difference of unweighted and weighted population (C), and rank of the p−value for pretreatment variables in the unweighted and weighted population (D).


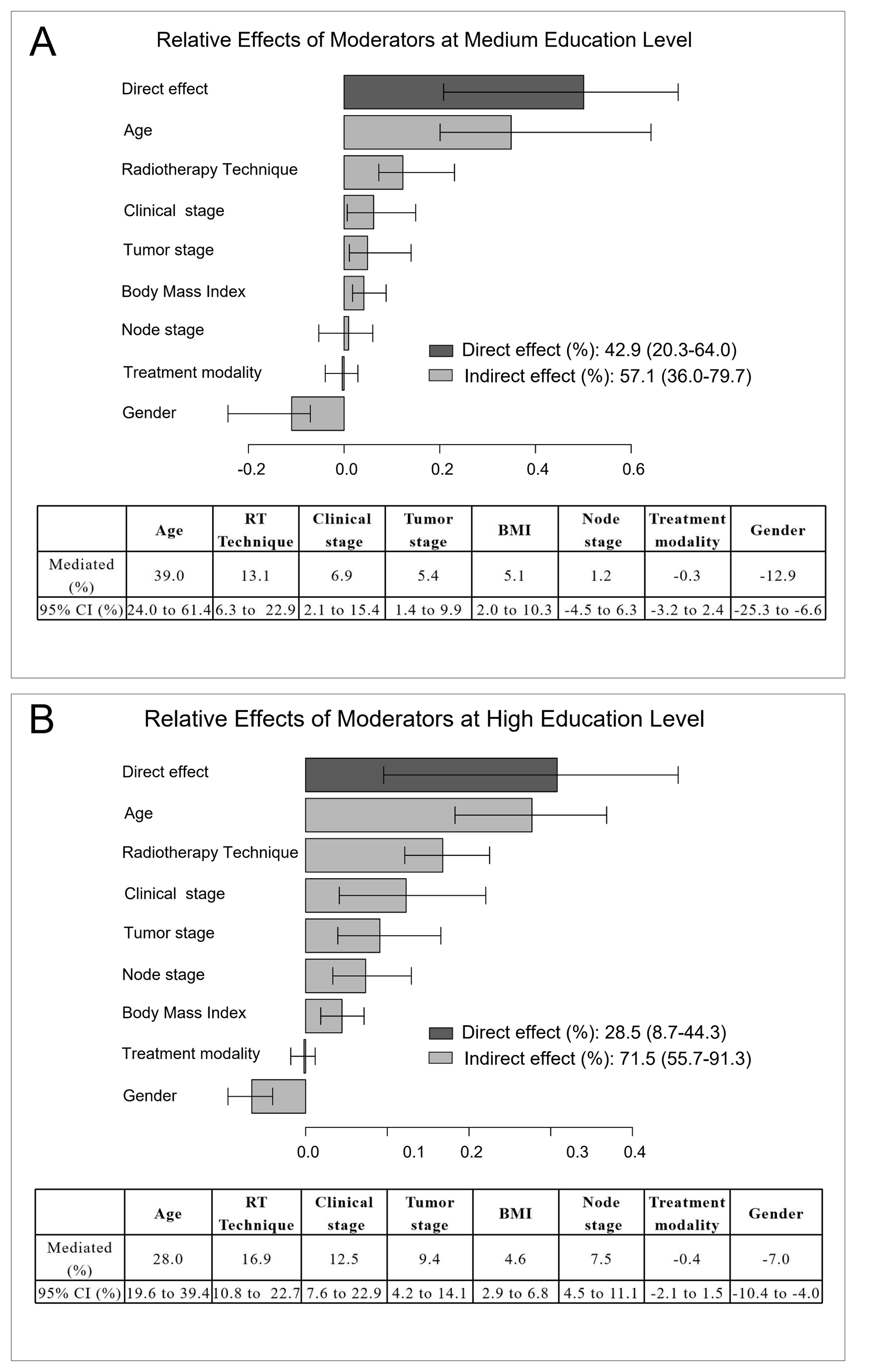


**Figure S2.** Relative effects of moderators in patients with medium (A) and high (B) education level.
